# Prevalence and determinants of stereotypic behaviours and physiological stress among tigers and leopards in Indian zoos

**DOI:** 10.1371/journal.pone.0174711

**Published:** 2017-04-17

**Authors:** Janice Vaz, Edward J. Narayan, R. Dileep Kumar, K. Thenmozhi, Krishnamoorthy Thiyagesan, Nagarajan Baskaran

**Affiliations:** 1 Department of Zoology & Wildlife Biology, A.V.C. College (Autonomous), Mannampandal, Mayiladuthurai, Tamil Nadu, India; 2 School of Science and Helath, Western Sydney University, Hawkesbury Campus, Richmond NSW, Australia; 3 Centre for Venom Informatics, Department of Computational Biology & Bio-informatics, Kariavattom North Campus, University of Kerala, Kerala, India; Centre for Cellular and Molecular Biology, INDIA

## Abstract

India’s charismatic wildlife species are facing immense pressure from anthropogenic-induced environmental perturbations. Zoos play a major role in the conservation of threatened species, but their adaptation in captivity is posing a major challenge globally. Stress from inadequate adaptation could lead to suppression of cognitive functioning and increased display of stereotypic behaviour. It is thus necessary to measure biological traits like behaviour, stress physiology, and contextual factors driving the animals maintained at zoos. In this study, we assessed stereotypic behaviour and stress physiology employing standard behaviour scoring, non-invasive stress monitoring, and their contextual drivers in a sub-population of two large felid species managed in six Indian zoos. The prevalence and intensity of stereotypic behaviours and levels of faecal corticosterone metabolites (FCM) were ascertained among 41 Royal Bengal tigers *Panthera tigris tigris* and 21 Indian leopards *Panthera pardus fusca* between April 2014 and March 2015. Behavioural observations showed that tigers spent more time stereotyping (12%) than leopards (7%) during daylight hours. Stress levels assessed using FCM revealed that tigers (23.6 ± 1.62 ng/g) had marginally lower level of corticosterone metabolites than leopards (27.2 ±1.36 ng/g). Stereotypic behaviour increased significantly with FCM level when the effect of heath status was controlled in tigers, and the effects tree cover, stone, den and keeper attitude controlled in leopards. Comparison of stereotypes of tigers with various biological and environmental factors using binary logistic regression revealed that stereotypic prevalence decreased with increased enclosure size, and enclosure enrichments like presence of pools and stones, when managed socially with conspecifics, and with positive keeper attitude, these factors accounting for 43% of variations in stereotypic prevalence among tigers. Stereotype among leopards was significantly absent when associated with increased tree cover and presence of pool, and den in the enclosure, age and among zoo-born than wild-born ones. These factors explain 81% of variations in stereotypic prevalence in them. A comparison of FCM levels with context-dependent factors revealed that stress levels among tigers decreased significantly with enclosure size and with individuals from nil to low, and severity of health issues. These factors explain 64% of variations in FCM levels. In leopards, the presence of stones in the enclosure and keepers with positive attitude resulted in significant decrease in FCM levels, these factors together accounting for 94% of variations. Multiple regressions on selected variables based on Factor Analysis of Mixed Data showed that in tigers the intensity of stereotype decreased significantly with enclosure size, sociality and positive keeper attitude and FCM level with health problems. Similarly, analyses in leopards revealed that intensity of stereotype decreased significantly with tree cover, age and FCM level with positive keeper attitude. Overall, our study suggests that to reduce stereotypes and stress level, tigers in captivity should be managed in larger enclosures enriched with pool, and stones, and in appropriate social conditions with adequate veterinary care. Leopards should be managed in enclosures with dense tree cover, pool, stones and den. Positive keeper attitude plays a crucial role in the welfare of both the species in captivity. Our study is promising and is comparable with their natural behaviour in the wild; for example, tigers require larger natural habitats, while leopards can manage even with smaller isolated patches but with dense vegetation cover.

## Introduction

Anthropogenic-induced environmental perturbations often impose immense pressure on Indian wildlife, especially on wide-ranging and charismatic species like tiger *Panthera tigris* [1–2]. In such a scenario, zoos would play a vital role in the conservation of such threatened species through captive breeding, research and education. However, many zoos around the world keep animals confined to small spaces compared to their wide-ranging peers in the wild. Due to spatial constraints the captive environments have difficulty in providing the ideal setting for their natural behaviour like hunting (the “hide, stalk and chase”) [3] resulting in welfare issues among captive animals [4]. Animal welfare science is a growing scientific discipline with great potential through which basic behavioural sciences are integrated with physiology, immunology and pathology to enable new-found knowledge to better the animals’ lives [5]. Animals in captivity exhibit abnormal behaviour due to poor welfare, since behaviour is an animal’s “first line of defence” in response to environmental change, i.e., what animals do to interact with, respond to, and control their environment [6]. Stereotypes have long been regarded as one such abnormal behaviours associated with sub-optimal environment for animals in captivity and indicators of stress due to poor welfare conditions of captive animals [7–8] any abnormal behaviour among captive animals should be addressed effectively. Stereotypic behaviour can be described as a pattern of movement such as pacing and head bobbing that is performed repeatedly, relatively invariant in form, and has no apparent function or goal [9]. Under captive condition, stereotypic behaviour is influenced by various contextual factors including elements of the enclosure [10–11], construction noise [12], animal care routines [13–16] and isolation from maternal relatives and conspecifics [17–19]. However, stereotypes may not correspond to current wellbeing alone, as they might be a ‘‘scar” from their previous suboptimal environments as well [20–21].

In mammals, another indicator of stress in captivity is the elevated level of glucocorticoids (cortisol and corticosterone) in blood and feces. Stress modulates the activities of the hypothalamo-pituitary-adrenal (HPA) axis and sympatho-adrenal axis releasing glucocorticoids or stress hormones to counter aversive stimuli [22–23]. Glucocorticoids released under stressful conditions help the organism defend itself against a perceived stressor [24]. Stress among captive animals is usually associated with elevated glucocorticoids [25–26, 9]. High glucocorticoid variability is considered an indicator of chronic or prolonged stress [27]. Non-invasive endocrinology techniques, such as monitoring of faecal glucocorticoid metabolites (FGM) in felids, especially in endangered species [28], have provided an ethically sound approach for quantifying physiological stress in animals under conservation management programmes [29]. They are easy to obtain [30], cause no disturbance to the study animals [30–31] and do not interfere in their natural behaviour due to capture or restraint as experienced in invasive sampling [32]. Such studies that use both rapid and reliable measures of stress hormones, and qualitative behavioural observations are likely to provide in-depth assessment on welfare condition of felids in captivity [33–34].

In general, animals in captivity have little or no control over the duration, and nature of light, sound, odours, visitors or temperatures to which they are exposed to. These variables influence animal activity or behaviour in captivity [4]. Environmental enrichment in suboptimal captive conditions may help reduce behavioural stress, which could lead to improved health, reproduction, and longevity [35]. It can be defined as an animal husbandry principle that seeks to enhance the quality of captive care by identifying and providing environmental stimuli necessary for optimal psychological and physiological wellbeing [36]. The size of the exhibit plays a large role in all enrichment programmes. Many exhibits are designed with the species’ natural history in mind and target the expression of species-typical behaviours as a goal [37].

In this study, we have evaluated, under captive conditions, the effects of biological and environmental factors on the stereotypic behaviour (as a psychological response to stress) and level of Faecal Corticosterone Metabolites (FCM) (as a physiological stress response) on two large felid species, viz., the Royal Bengal tiger (*Panthera tigris tigris*) [38] and Indian leopard (*Panthera pardus fusca*) [38], housed in six Indian zoos. The Royal Bengal tiger (*P*. *tigris tigris*), a sub-species of *Panthera tigris*, is an endangered species (IUCN Red List 2016) with an estimated population of 2200 individuals in the wild [39], and 367 individuals in Indian zoos [40]. The Indian leopard (*P*. *pardus fusca*), a sub-species of *Panthera pardus* [41], has been declared as near-threatened (IUCN Red List, 2016). In 2014, the first-ever scientific national census of leopards around tiger habitats in India, excluding the northeast, estimated its number as 7910 individuals, with a speculated national total of 12,000–14,000 [42] and 431 individuals managed in Indian zoos [40]. Both these cats come under Schedule I category of the Indian Wildlife Protection Act, 1972 [43]. The findings of this study have important implications for efficient management of wild and captive populations of felids and on their reintroductions, which has as yet received little attention [44]. The objectives and corresponding hypotheses of this study are as follows:

Objective 1: To evaluate the intensity of stereotype among the focal tigers and leopards managed in six different zoos in India.
Hypothesis 1: Individual tigers and leopards would vary in their stereotypic behaviour depending on their biological (origin, sex, age, reproductive and heath condition) and environmental factors (ambient temperature, enclosure and its enrichments, keepers’ attitude and visitor number) in captivity.
Objective 2: To quantify the levels of FCM of tigers and leopards of the study sites.
Hypothesis 2: Mean level of FCM would vary among individual tigers and leopards in relation to their biological and environmental factors prevailing among the study subjects and sites (as mentioned above).
Objective 3: To relate the extent of stereotypic behaviour exhibited by individual tigers and leopards with their respective level of FCM recorded.
Hypothesis 3: Extent of stereotypic behaviour among individual tigers and leopards would vary with the level of FCM.
Objective 4: To identify the factors influencing the appearance and extent of stereotypic behaviour and FCM in the study species under captive conditions.
Hypothesis 4: Evaluation of the association of both biological and environmental factors in captive conditions with the appearance and extent of stereotypic behaviour and the level of FCM of tigers and leopards would reveal the individual factor level (significant/insignificant) and nature (positive/negative) of influence on the stereotypes and FCM levels.

## Materials and methods

### Ethics statement

All observations and faecal sample collection procedures were as per the relevant National Guidelines. Permission was obtained from the directors of the respective zoos, who evaluated and approved our observation procedures and non-invasive faecal sample collections as part of granting field permits prior to the starting of the work. The study subjects were observed only during their routine husbandry practices and no experiments were conducted. Non-invasive method (faecal sample) was employed in this study to assess the physiological stress. The faecal samples were collected while the study animals were away from the defecated location (indoor or outdoor enclosures). Since the vertebrate research work was approved by the respective zoos and did not involve handling of animals or experimentation or collection of blood or tissue samples, there was no need for approval from Institutional Animal Care and Use Committee (IACUC) or equivalent animal ethics committee. The zoos that approved our study were: (1) Rajiv Gandhi Zoological Park and Wildlife Rescue Centre, Pune, (2) Animal Rescue and Rehabilitation Centre, Pune, (3) Thiruvananthapuram Zoo, Thiruvananthapuram (4) Thrissur Zoo, Thrissur, (5) Arignar Anna Zoological Park, Chennai, and (6) National Zoological Park, Delhi.

### Study site and species

The study involved sampling of 41 Royal Bengal tigers, and 21 Indian leopards housed at six different zoos in India mentioned above and was carried out over a period of seven months from April to May 2014, and from October 2014 to March 2015 (**Table 1**). Animals that were managed for less than two months in new abodes and with major health issues such as nervous disorder, partial paralysis were excluded from the study. The cats were housed either singly, or in groups. Zoos that have a single outdoor exhibit but with more than one cat, let them out in turns. Every zoo had one main keeper and one or more assistant keepers for each cat. Most zoos have a single veterinarian for the entire zoo. The study subjects were let out into the outdoor exhibit enclosure from 09:00 to 18:30 h. Beef and/or chicken are served to them daily between 14:00 and 18:00 h. All the zoos are shut once a week for cleaning. Observations began from 9:00 h, when the zoos were opened, and the cats were let into the outdoor enclosures, and lasted till 18:30 h, when they are closed and the cats transferred back to night cells.

**Table 1:** Details of various study sites and study species.

Study site	Study Period	Temperature (C°)	Tiger			Leopard		
			♂	♀	Total	♂	♀	Total
Rajiv Gandhi Zoological Park (18°27’14.3”N and 73°51’31.67”E)	Apr.—May 2014	34.4	2	3	5	1	2	3
Number of enclosures used					2			1
Animal Rescue and Rehabilitation Centre, Pune	May 2014	31.6	0	0	0	1	3	4
(18°27’18.92”N and 73°51’33.10”E)								
Number of enclosures used					0			3
Thiruvananthapuram Zoo	Oct.—Dec. 2014	28.9	4	3	7	2	3	5
(8°30’41.82”N and 76°51’17.80”E)								
Number of enclosures used					6			2
Thrissur Zoo	Jan. 2015	29.3	2	1	3	3	1	4
(10°31’48.96”N and 76°13’22.53”E)								
Number of enclosures used					2			4
Arignar Anna Zoological Park	Feb. 2015	27.9	8	10	18	2	1	3
(12°52’41.08”N and 80°5’90.13”E)								
Number of enclosures used					3			2
National Zoological Park	Mar. 2015	24.3	5	3	8	1	1	2
(28°36’6.84”N and 77°14’51.72”E)								
Number of enclosures used					3			2

### Evaluation of the intensity of stereotype

Details such as age-sex of individual cats, their origin, reproductive and health history were obtained from the register of records and studbook database called ‘ZIMS’ available with the zoos. Individual cats were categorised into three different major age classes—young age (1–5 years), middle age (6–15 years), and old age (>15 years). We sampled 41 tigers for stereotype, of which 17 belonged to young age category, 21 were middle aged, and three old tigers. Similarly, we sampled stereotype among 21 leopards, of which five were young, 10 middle aged and six old (see supplementary material for more details). The individuals were categorised as zoo or wild born based on their birth location and their reproductive status as bred or not bred, based on individual cat mating and birth records of cubs. Their health issues of the present and the past were considered and rated 0 for no health problem, 1 for low (wounds caused by infighting), and 2 for high (any major surgery).

Focal sampling method was employed to assess stereotypic behaviour [45]. All target individuals were observed for a minimum of three days from 09:30 to 17:00 h at 15-min interval with 10-min observation and five minutes rest. During observation, various activities exhibited by the study subjects were recorded to the nearest second, and an ethogram of different behaviours was prepared, including resting, feeding, moving, stereotyping, playing and others, which include all the remaining activities such as grooming, scratching, fighting, staring, Flehmens’ response and mating.

### Sampling scat for the extraction of faecal glucocorticoids metabolite

In total, 62 fresh faecal (scat) samples were collected, from 18 tigers (n = 41) and nine leopards (n = 21) managed at three study zoos—Thiruvananthapuram Zoo, Thrissur Zoo and Arignar Anna Zoological Park—during the behavioural sampling period. Of the 41 scat samples from tigers, 21 were from males and 20 from females, and of the 21 from leopards, 10 were from males, and 11 from females. The 18 tigers sampled for scat collection included six young ones, 11 middle-aged ones and one old aged. Of the nine leopards sampled, scats were collected from three individuals each in young, middle and old-age classes. The faecal samples were collected based on visitor intensity (low on week days and high on weekends). The faecal samples were collected two days after the behavioural observations either immediately or within an hour of defecation. The entire scat was collected, homogenized it thoroughly and taken a sample from it as the metabolites may not be distributed in the scat equally. To avoid contamination hand gloves and sterile scalpel were used to collect the faecal samples in 5ml sterile centrifuge tubes. The collected samples were frozen in liquid nitrogen or dry ice in an ice box to maintain the integrity of the steroid metabolites [46]. They were transferred later to the Centre for Venom Informatics Laboratory, University of Kerala, and stored for two months in a freezer at -20°C prior to assay.

### Extraction of faecal glucocorticoid metabolites—Corticosterone

Faecal glucocorticoid metabolites were extracted by slight modification of the methods used for clouded leopard *Neofelis nebulosa* [47], Sumatran tigers [48], and Royal Bengal tigers [49]. Samples were dried in a lyophiliser and sieved to get a homogenised powdered hair-free sample. A sample of 0.05 g was added to 1 mL of 80% methanol, vortexed for 30 min and centrifuged at 2500 rpm for 20 min in a refrigerator centrifuge. Methanol was allowed to evaporate from the sample using liquid nitrogen. The dried samples were suspended in 500 μL of the enzyme-immunoassay (EIA) buffer, with 1 M phosphate solution containing 1% BSA, 4 M sodium chloride, 10 mM EDTA and 0.1% sodium azide with a pH of 7.5.

### Faecal glucocorticoid metabolite EIA

The EIA kit suitable for measuring glucocorticoid metabolites in animal faeces was procured from Cayman Chemicals (USA). It contained polyclonal corticosterone EIA antiserum, corticosterone EIA AChE tracer, corticosterone EIA standard, EIA buffer concentrate, wash buffer concentrate, polysorbate 20, rabbit anti-sheep IgG-coated 96 well EIA plate, 96 well cover sheet, and Ellman’s reagent. The EIA buffer was diluted with 90 ml of ultra-pure water. For the wash buffer, the 5 ml vial was diluted to 2 L and 1 ml polysorbate 20. Using the tracer, the 100 determinations (dtn) vial was reconstituted with 6 ml of EIA buffer for the corticosterone antiserum. Standards were prepared by serial dilution process. The reagents were added to the 96 well EIA plate provided with the kit. The plate was covered and incubated for two hours at room temperature with gentle shaking, washed further and the tracer was added with Ellman’s reagent and incubated for a further 90 min (av.) at room temperature with gentle manual shaking. The plate was read on a microplate reader—iMark Microplate Absorbance Reader (*BIORAD*) at 420 nm wavelength. Intra- and inter-assay coefficients of variation were 6.075% (*n* = 16, triplicates of single sample) and 5.98% (*n* = 47 assays), respectively. Sensitivity of the assay at maximum binding was 150 pg/ml and the limit of detection was 30 pg/ml. FCM concentrations were expressed as nano grams (ng) per gram (g) of dry faecal matter (ng/g).

### Assessing the environmental characteristics and their influence on stereotype and faecal glucocorticoid metabolites

Environmental variables such as enclosure size, enrichment factors, ambient temperature, visitor disturbance and keepers' attitude towards the animals that they handle were measured for each study subject. To assess the current enrichment items, the enclosure size was either measured or the details obtained from the zoo records. The enclosures were categorised as small or large based on the guidelines of Central Zoo Authority of India [50], which suggests 1000 m^2^ minimum space of outdoor enclosure per pair of tigers and 500 m^2^ per pair of leopards for captive animal management in India. Other enclosure enrichment factors assessed include: the substrate type (natural/artificial), vegetation (i.e., grass and tree cover: % rating by ocular method), and the pool, den or hide, logs, natural barriers and artificial thermoregulators (qualitatively as present/absent). Ambient temperature was recorded at hourly intervals during focal observation time using a digital thermometer. Data on visitor number was obtained from the zoo records. The visitor days were categorised as no visitor (zoo holiday), low visitor (week days) and high visitor days (weekends). The keepers’ attitude was categorised as neutral for keepers who spent moderate time and had neutral interaction with his animal (coded as 0), positive for keepers who spent maximum time with caring interaction (coded as 1), and negative for keepers who spent minimum time with harmful interaction (coded as 2) that could irritate or injure the animal. The attitude of the keeper was assessed based on observations over a period of time.

### Statistical analysis

Statistical analysis was made using SPSS for Windows (*Version 21*), developed by SPSS Inc. and Software R (*Version 13*.*2*) for Principal Component analysis. Data were checked for homogeneity of variance and normality prior to detailed analysis. The data on stereotypic behaviour and FCM level, the dependent factors, of both tigers and leopards did not show homogeneity of variance and normality, so the variables were transformed using options such as log, square route, arcsine and inverse transformations. Among the four, log transformations fulfilled homogeneity and normality requirements for stereotype and FCM among tigers, and only for stereotype among leopards. Data on FCM of leopard did not normalise with any of the four options. Therefore, the data on stereotype of tigers and leopards and of FCM of tigers were analysed using parametric statistical tests, while the FCM data of leopards was analysed using nonparametric statistical analysis. The level of significance, α, was set at 0.05. Differences in the levels of stereotype and FCM due to various biological and environmental variables among tigers and stereotype alone for leopards were tested using Independent-samples T-test (for variables with two categories) or Univariate Analysis of Variance (for variables with three categories). Since the FCM data of leopards could not be normalized, it was tested using non-parametric tests, viz., Mann–Whitney U-test (for variables with two categories) or Kruskal–Wallis One-Way ANOVA (for variables with three categories) [51].

MANOVA was used to test the differences between categories in different biological and environmental variables (treated as independent variables) in the present study across stereotypic behaviours and FCM (treated as dependent variables). Box's test was used to test the assumption of equal covariance matrices. Pillai's Trace was used as a test of statistics to assess if the groups (categories) differ significantly with respect to dependent variables. Significant MANOVA was followed with Univariate ANOVA and Discriminant Function Analysis (DFA) to see how the dependent variables discriminate the groups. DFA identifies variates (the combination of the dependent variables) and Wilk's Lambda Values were used to ascertain how many variates are significant. If the values of significance are < 0.05, the variate is significant in discriminating the groups. Once the significant variables are identified, the Standardised Canonical Discriminant Function Coefficients are used to find out how different variables contribute to the variates, with high score indicating the importance of a dependent variable for a variate. Variables with a positive and negative coefficient contribute to the variate in opposite ways. The Canonical variate correlation coefficients are used to find out the relative contributions of each dependent variable to group separation and the relative contributions of each variable to the variate. The group centroids show which group is discriminated by a variate. The discriminant scores are plotted to show the group separations.

### Relationship between stereotypic behaviour and FCM levels

To understand the influence of FCM on stereotype, the extent of stereotypic behaviour of individual tigers and leopards were compared with the respective FCM levels using simple regression analysis treating FCM as independent variable and stereotype as dependent variable. Further, the confounding effects of biological and environmental variables, if any, on the relationship between stereotype and FCM, were analysed using ANCOVA.

### Binary logistic regression

We used binary logistic regression to ascertain the subset of the environmental (enclosure size and the other environmental factors including keeper attitude and visitor number) and biological (origin, age, sex, sociality, breeding and health status) variables that contribute to the onset of stereotypes and higher level of FCM. The binary logistic regression is essentially used for qualitative data (presence/absence type of environmental factors), and the same principle is extended here to learn the factors that promote the appearance or the onset of stereotype and low or high level of FCM among the study subjects. For the stereotypic behaviour, the data inputs are coded as 0 for absence and 1 for presence, and for FCM, 0 for low and 1 for high levels. As the normal or baseline levels of FCM are not available in the literature [28] for coding, they were calculated as follows: at first, mean plus two standard deviations of the total concentrations of metabolites of an individual was calculated. This level represent the limit. The values above the limit were eliminated and the calculations were repeated until all values were below the limit. Later their mean value was obtained to get the baseline value. The baseline values so obtained were 22.753 ng/g for tigers, and 26.601 ng/g for leopards. However, the values are applicable only for the present dataset and need to be standardised with larger dataset from varied biological and environmental conditions for universal application.

### Multiple regression

Binary logistic regression reveals conditions qualitatively the presence/ absence of stereotypes and low or high levels of FCM. We used multiple regression analysis to assess the quantitative effects of the biological (age, sex, origin, sociality, reproductive status, and health problems) and environmental variables (enclosure size and its enrichment factors like tree cover, grass cover, logs, pools, keepers' attitude and visitor number) on the extent or intensity of stereotypic behaviours and FCM levels among the study animals. The regression was performed in two steps. Step 1: At first, "Principal Component Analysis was performed using Factor Analysis Mixed Data" (FAMD) [52] option (as our datasets consist of both continuous and categorical variables) in 'FactoMineR' package [53] of software R (*Version 13*.*2*). The variables that had high COS^2^ with the first three components for stereotype and the first two components for FCM were considered for multiple regression analysis. This step reduces the number of variables used in the multiple regression equations. Step 2: The variables so obtained were taken into multiple regression analysis using SPSS to get a subset of variables that best predict the effects of dependent variables, viz., stereotype and FCM levels. The binary logistic regression analysis identified variables that resulted in the onset of stereotype and high level of FCM; multiple regression analysis gave the variables that enhance the levels of stereotype and FCM among the study subjects.

## Results

### (1) Overall prevalence and extent of stereotypes and FCM levels

Of the 41 tigers and 21 leopards observed, 83% of tigers and 62% of leopards exhibited stereotypic behaviour. In total, the two study species displayed four different types of stereotypic behaviours, viz., repetitive walk or trot, head rotation, chewing paws and snapping. While the stereotypic extent was higher among tigers (14.6 ±1.63) than leopards (5.90 ±1.64), the FCM level was lower among tigers (23.6 ±1.62) than leopards (27.2 ±1.36).

### (2) Stereotypic behaviour and FCM level in relation to biological factors

When the origin of the felids, wild- or captive/zoo-born, is considered, the zoo-born tigers stereotyped significantly higher (*t* = 2.58; p = 0.01) than the wild-born ones, while in leopards, the wild-born individuals stereotyped significantly higher (*t* = 2.20; p = 0.03) than the captive-born ones (**Table 2**). Between the sexes, males stereotyped numerically higher than females in both tigers and leopards, but the difference was statistically significant only in leopards (*t* = 2.6; p = 0.02). Among the age-classes, the middle-aged tigers showed significantly more stereotype than the young and old age-classes (*F* = 3.41; p = 0.04), while in leopards, young age-class showed significantly more stereotype than middle and old aged (*F* = 2.29; p = 0.03). Tigers bred in the past stereotyped numerically higher than the unbred ones, but the difference was not statistically significant (*t* = 0.09; p = 0.93), while in leopards, the unbred ones stereotyped significantly higher than the bred ones (t = 2.28; p = 0.03). Both tigers and leopards showed more stereotype when managed in solitary condition compared to social condition, i.e., with their conspecifics (tiger: *t* = 2.62; p = 0.01, leopard *t* = 4.17; p = 0.000). Among the tigers, the frequency of stereotype was significantly higher while facing health issues (*F* = 14.37; p = 0.000), but the effect of health status on stereotype was not significant among leopards (*F* = 1.865; p = 0.18). On the other hand, the FCM levels did not vary on the basis of origin, sex, age-classes, breeding status and health problems (p > 0.05) both in tigers and leopards

**Table 2:** Extent of stereotypic behaviour and FCM (±SE) in relation to biological factors in tigers (*n* = 41) and leopards (*n* = 21). Note: *F* indicates Univariate Analysis of Variance; *t* indicates independent-samples T test, *U* indicates Mann-Whitney U test and *χ*^*2*^ = Kruskal-Wallis test.

Attributes	Categories	Stereotypic Behaviour (%/h)		FCM (ng/g)	
		Tiger (*n*)	Leopard (*n*)	Tiger (*n*)	Leopard (*n*)
Origin	Wild born	6.5 ± 1.35 (16)	7.2 ± 1.37 (38)	24.4 ± 3.48 (04)	27.6 ± 2.19 (10)
	Zoo born	16.2± 1.38 (98)	1.7 ± 1.02 (15)	23.5 ± 1.76 (39)	26.9 ± 1.74 (10)
	Test & (p) value	*t* = 2.58 (0.01)	*t* = 2.20 (0.03)	*t* = 5.17 (0.61)	*U* = 47 (0.82)
Sex	Male	17.9 ± 1.83 (60)	10.2 ± 1.99 (25)	19.5 ± 1.58 (27)	27.3 ± 2.24 (10)
	Female	11.3 ± 1.53 (54)	1.6 ± 0.50 (28)	30.4 ± 2.76 (16)	27.1 ± 1.68 (10)
	Test & (p) value	*t* = 0.63 (0.53)	*t* = 2.6 (0.02)	*t* = 1.17 (0.26)	*U* = 47 (0.82)
Age	Young	8.2±1.54 (47)	13.7± 4.18(13)	29.0±4.07 (15)	26.8±2.82 (6)
	Middle	21.0±2.57 (60)	4.9± 2.31(26)	20.9±0.89 (27)	26.2± 0.94 (7)
	Old	4.4±1.88 (7)	0.48±0.48 (14)	14.2 (1)	27.3±1.36 (7)
	Test & (p) value	*F* = 3.41 (0.04)	*F* = 2.95 (0.03)	*F* = 2.82 (0.75)	*χ*^*2*^ = 0.62 (0.73)
Breeding	Bred	17.3 ± 2.77 (24)	2.9 ± 1.08 (29)	21.5 ± 2.35 (11)	28.0 ± 1.81 (14)
	Not bred	14.1 ± 1.35 (90)	8.9 ± 1.79 (24)	24.2 ± 2.0 (32)	26.1 ± 2.13 (6)
	Test & (p) value	*t* = 0.09 (0.93)	*t* = 2.28 (0.03)	*t* = 0.94 (0.36)	*U* = 41 (0.93)
Sociality	Solitary	19.0 ± 2.25 (75)	9.5 ± 2.72 (30)	21.0 ±1.55 (28)	30.5 ±2.49 (9)
	Social	6.5 ± 1.15 (39)	1.2 ± 0.51 (23)	28.4±3.37 (15)	24.6 ±0.87 (11)
	Test & (p) value	*t =* 2.62 (0.01)	*t* = 4.17 (0.000)	*t =* 0.03 (0.09)	*U* = 30 (0.14)
Health problem	No	6.3 ± 0.84 (55)	8.1 ± 1.93 (13)	47.0 ± 0.64 (10)	27.7 ± 2.64 (5)
	Low	13.6 ± 2.55 (20)	4.1 ± 1.92 (24)	21.1 ± 2.47 (12)	29.0 ± 3.01 (7)
	High	27.3 ±2.69 (3	5.9 ± 2.25 (16)	22.2 ± 1.65 (21)	25.1 ± 1.20 (8)
	Test & (p) value	*F* = 14.37 (0.000)	*F* = 1.865 (0.18)	*F* = 0.927(0.42)	*χ*^*2*^ = 0.41(0.82)

### (3) Stereotypic behaviour and FCM level in relation to environmental factors

Among the 12 environmental factors assessed, stereotypic behaviour of tiger varied significantly in relation to 10 factors (**Table 3**). These include percent grass (*t* = 4.81; p < 0.001) and tree (*t* = 4.81; p < 0.001) cover in the enclosures (tigers in enclosures with higher grass and tree cover stereotyped significantly lower than those in enclosures with lower cover), nature of enclosure substrates (tigers in enclosures with natural substrate stereotyped significantly in lower level than those in artificial substrates (*t* = 3.73; p < 0.001)), enrichments, viz., presence of stones (*t* = 7.30; p < 0.001), den (*t* = 3.72; p < 0.001), temperature regulators (*t* = 2.36; p = 0.02), and barriers (*t* = 3.21; p = 0.002) in the enclosure (tigers in enclosures with presence of enrichments had significantly lower level of stereotype than those in their absence), keepers’ attitude (tigers kept under keepers with positive attitude stereotyped significantly lower than those kept under keepers with neutral and negative attitudes (*F* = 17.5; p < 0.001)), visitor number (tigers during higher visitor days stereotyped significantly lower than lower and nil visitor days (*F* = 3.44; p = 0.03), and enclosure size (tigers in larger enclosures have significantly lower stereotype than those in smaller enclosures (*t* = 5.42; p = 0.000)). On the other hand, in leopards, stereotypic behaviour varied significantly with only five out of eleven factors, viz., percent grass (*t* = 4.26; p = 0.000) and tree (*t* = 4.01; p = 0.000) cover in the enclosures (leopards in enclosures with lower grass and tree cover stereotyped significantly higher than those in enclosures with higher grass and tree cover), nature of enclosure substrates (leopards in enclosures with natural substrates stereotyped significantly lesser than those in artificial substrates (*t* = 3.25; p = 0.01)), and enclosures with the presence of stones (*t* = 3.24; p = 0.003) and den (*t* = 2.70; p = 0.01) result in lower stereotype than in enclosures with the absence of these enrichments.

**Table 3:** Extent of stereotypic behaviour and FCM (with± S.E) in relation to environmental factors in tigers (*n* = 41) and leopards (*n* = 21). Note: *F* indicates Univariate Analysis of Variance; *t* indicates independent-samples T test, and *U* indicates Mann-Whitney U-test.

Attributes	Categories	Stereotypic behaviour (time spent/h)		FCM (ng/g)	
		Tiger (*n*)	Leopard (*n*)	Tiger (*n*)	Leopard (*n*)
Ambient temperature	Low	10.2 ± 3.16 (13)	12.8 ± 7.09 (6)	- (0)	- (0)
	Medium	16.4 ± 2.04 (86)	6.5 ± 2.32 (32)	24.2 ± 2.99 (17)	27.3 ± 1.97 (11)
	High	9.1 ± 2.53 (15)	1.9 ± 0.55 (15)	17.7 ± 2.21 (5)	26.2 ± 2.92 (5)
	Test & (p) value	*F* = 2.63 (0.07)	*F* = 3.08 (0.06)	*t* = 0.693 (0.49)	*U* = 21.0 (0.46)
Grass cover (%)	Low	26.08 ±3.43 (35)	9.43 ± 2.73 (30)	20.92 ± 1.04 (20)	30.3 ± 2.02 (11)
	High	9.7 ±1.47 (79)	1.30 ± 0.51 (23)	25.9 ± 2.84 (23)	23.49± 0.59 (9)
	Test & (p) value	*t* = 4.81(0.000)	*t* = 4.26 (0.000)	*t* = 0.016 (0.99)	*U* = 30.0 (0.14)
Tree cover (%)	Low	26.1±3.43 (35)	9.76 ± 2.80 (29)	20.9 ± 1.04 (20)	30.3 ± 2.02 (11)
	High	9.7±1.48 (79)	1.24 ± 0.45 (24)	25.9 ± 2.84 (23)	23.5 ± 0.59 (9)
	Test & (p) value	*t* = 4.82 (0.000)	*t* = 4.01(0.000)	*t* = 0.169 (0.87)	*U* = 30.0 (0.14)
Substrate	Artificial	20.3 ± 2.49 (56)	16.9 ± 5.57 (11)	21.9 ± 2.06 (18)	27.1 ± 2.74 (6)
	Natural	9.3±1.86 (58)	3.01 ± 1.17 (42)	24.8 ± 2.37 (25)	27.3 ± 1.62 (14)
	Test & (p) value	*t* = 3.73 (0.000)	*t* = 3.25 (0.01)	*t* = 0.17 (0.87)	*U* = 36.0 (0.62)
Pool	Present	14.7±1.74 (104)	1.8 ± 1.01 (6)	24.2 ± 1.76 (39)	- (0)
	Absent	15.3±4.36 (10)	6.4 ± 1.84 (47)	17.2 ± 0.26 (4)	27.2 ± 1.36 (20)
	Test & (p) value	*t* = 8.61 (0.39)	*t* = 0.60 (0.55)	*t* = 0.53 (0.60)	- (0)
Stones	Present	10.5±1.43 (91)	3.6 ± 1.26 (39)	25.1 ± 2.10 (32)	24.6 ± 0.80 (12)
	Absent	31.2±4.27 (23)	12.2 ± 4.89 (14)	19.0 ± 0.75 (11)	31.2 ± 2.72 (8)
	Test & (p) value	*t* = 7.30 (0.000)	*t* = 3.24 (0.003)	*t* = 0.17 (0.87)	*U* = 30.0 (0.16)
Den	Present	10.7±1.58 (82)	3.7 ± 1.32 (37)	25.2 ± 2.41 (28)	25.7 ± 1.23 (15)
	Absent	24.9±3.58 (32)	10.9 ± 4.36 (16)	20.5 ± 0.88 (15)	31.8 ± 3.53 (5)
	Test & (p) value	*t* = 3.72(0.000)	*t* = 2.70 (0.01)	*t* = 0.35 (0.73)	*U* = 19.0 (0.106)
Temperature regulator	Present	9.0±2.34 (27)	- (0)	- (0)	- (0)
	Absent	16.5±1.97 (87)	- (0)	23.6 ±1.62 (43)	- (0)
	Test & (p) value	*t* = 2.356 (0.02)			
Barriers	Present	7.3±2.59 (20)	6.5 ± 3.05 (15)	- (0)	- (0)
	Absent	16.3±1.86 (94)	5.7 ± 1.98 (38)	- (0)	- (0)
	Test & (p) value	*t* = 3.21(0.002)	*t* = 0.36 (0.72)		
Keepers’ attitude	Neutral	24.3 ± 2.84 (49)	6.5 ± 3.88 (12)	20.3 ± 0.95 (15)	37.4 ± 1.12 (3)
	Positive	6.5 ± 1.18 (60)	5.7 ± 1.83 (41)	25.3 ± 2.39 (28)	25.5 ± 1.11 (17)
	Negative	19.6 ± 8.08 (5)	- (0)	- (0)	- (0)
	Test & (p) value	*t* = 17.5 (0.000)	*t* = 1.25 (0.22)	*t* = 0.17 (0.87)	*U* = 0.00 (0.007)
	No	21.6 ± 4.57 (11)	3.3 ± 0.98 (11)	26.5 ± 0.56 (2)	24.1 (1)
Visitors number	Low	16.8 ± 1.85 (61)	5.3 ± 2.49 (22)	21.7 ± 2.11 (23)	26.1 ±1.72 (12)
	High	10.1 ± 1.41(42)	8.0 ± 3.38 (20)	25.7 ± 2.75 (18)	29.8 ± 2.42 (7)
	Test & (p) value	*F* = 3.44 (0.03)	*F* = 0.31 (0.73)	*F* = 1.006 (0.37)	*U* = 26.0 (0.176)
Season	Summer	13.9 ± 1.20 (87)	6.0 ± 2.36 (23)	27.4 ± 2.94 (21)	28.9 ± 7.17 (2)
	Winter	17.2 ± 2.46 (27)	5.8 ± 2.31 (30)	19.9 ± 1.04 (22)	27.1 ± 1.40 (18)
	Test & (p) value	*t* = 1.34 (0.182)	*t* = 0.833 (0.41)	*t* = 1.801 (0.79)	*U* = 17.0 (0.90)
Enclosure size	Large	6.25± 0.41 (64)	1.85± 1.009(6)	11.8±0.62 (16)	27.3±1.36 (20)
	Small	18.61± 0.37(50)	6.4 ±1.84 (47)	22.2±0.37 (27)	- (0)
	Test & (p) value	*t* = 5.42 (0.000)	*t* = 0.60 (0.55)	*t* = 3.45 (0.003)	

Among the 10 environmental factors assessed, FCM level in tigers varied significantly only with enclosure size (tigers in larger enclosures have significantly lower FCM level than those in smaller enclosures (*t* = 3.45; p = 0.003; **Table 3**), while in leopards, it varied significantly only with keeper’s attitude (leopards under keeper with positive attitude had lower FCM level than those under keeper with neutral attitude (Mann-Whitney *U* = 0.000; p = 0.007)).

### (4) MANOVA using stereotype and FCM of tigers

Pillai's Trace Values (V) showed that variations due to health condition, % tree cover, nature of substrates, presence of stones, keeper attitude and enclosure size were significant (p < 0.05). All other biological and environmental factors had no significant role (p > 0.05; **Table 4**).

**Table 4:** MANOVA using stereotypic behaviour and FCM level on various biological and environmental variables among tigers in the study.

Variable	Pillai's Trace (Value)	F	df	p
Origin	0.070	0.561	2, 15	0.582
Sex	0.121	1.037	2, 15	0.379
Age-class	0.221	2.131	2, 15	0.153
Breeding history	0.202	1.902	2, 15	0.184
Sociality	0.026	0.200	2, 15	0.821
Health problem	0.601	3.222	4, 30	0.026
Ambient temperature	0.138	1.202	2, 15	0.328
Grass cover	0.262	2.666	2, 15	0.102
Tree cover	0.429	5.632	2, 15	0.015
Nature of substrate	0.429	5.632	2, 15	0.015
Pool	0.077	0.628	2, 15	0.547
Stone	0.429	5.632	2, 15	0.015
Den	0.296	3.157	2, 15	0.072
Keepers' attitude	0.429	5.632	2, 15	0.015
Visitor number	0.029	0.226	2, 15	0.801
Enclosure size	0.604	11.421	2, 15	0.001

**Fig 1:**
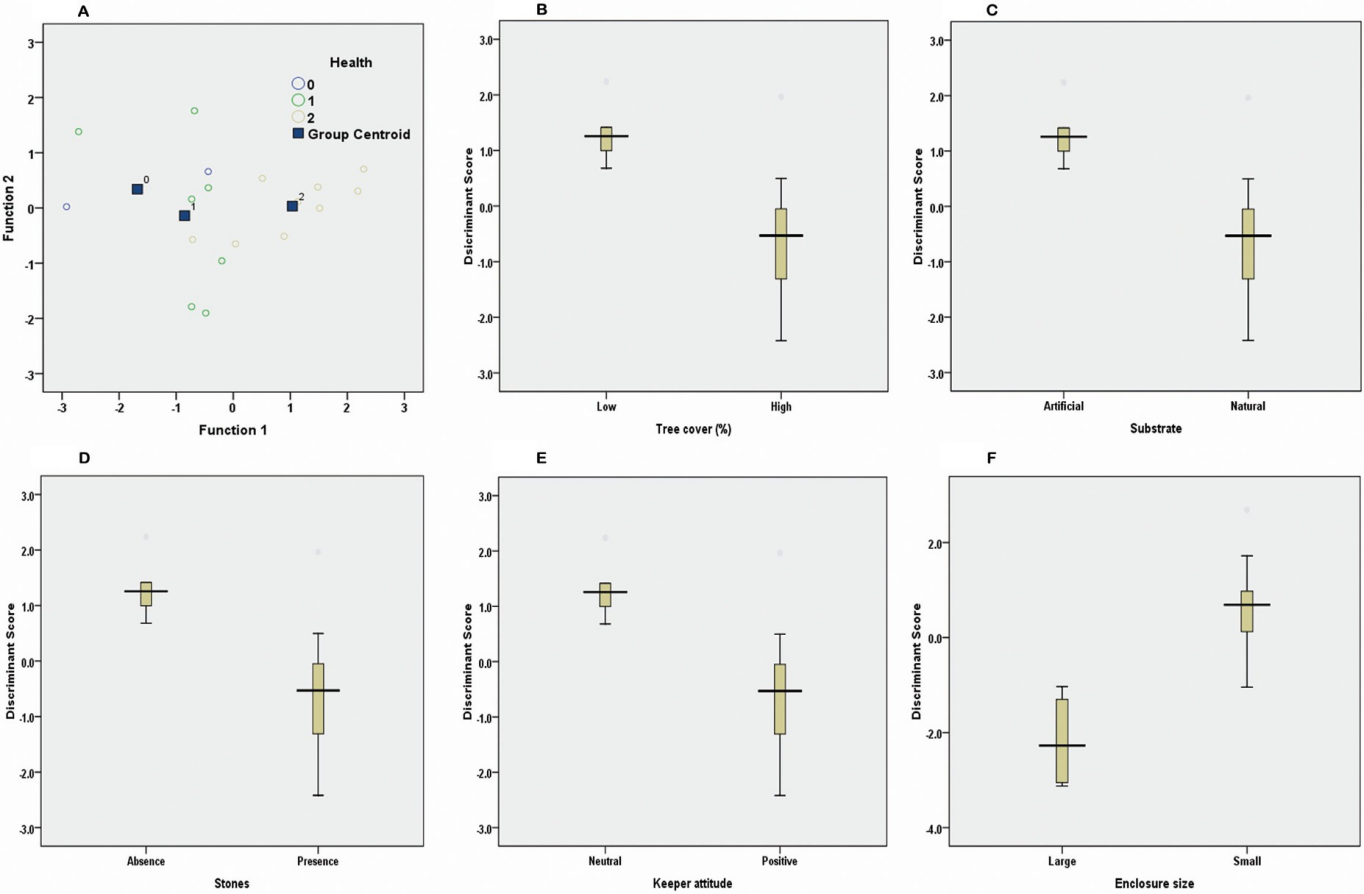
Plots of Canonical discriminant scores obtained from DFA to differentiate variables (1A: Health, 1B: Tree cover, 1C: Substrate, 1D: Stone, 1E: Keeper attitude and 1F: Enclousre size) that show significance in MANOVA.

Pillai’s Trace showed a significant effect of the health condition of the felids based on the frequency of stereotype and FCM levels (v = 0.601; F (4, 30) = 3.222; p = 0.026). However, a separate univariate ANOVA on the outcome variables revealed non-significant treatment effect on the frequency of stereotype (F (2, 15) = 1.965; p = 0.175) and FCM level (F (2, 15) = 0.927; p = 0.417). MANOVA followed by discriminate analysis revealed two discriminate functions. The first explained 98.2% of the variance, and Canonical R^2^ = 0.759, whereas the second explained only 1.8%, and Canonical R^2^ = 0.157. In combination, these two discriminate functions differentiated the health categories, Ʌ = 0.413, *χ*^*2*^ (4) = 12.812, p = 0.012, but the elimination of the first function did not significantly differentiate the second function, i.e., health category Ʌ = 0.975, χ^2^ (1) = 0.363, p = 0.547. The correlations between outcomes and discriminant functions revealed that the frequency of stereotype loaded fairly evenly with both the functions (r = 0.421 for the first functions, and r = 0.907 for the second). The FCM levels loaded more highly on the second functions (r = 0.962) than on the first ones (r = -0.271). The discriminant function plot showed that the first function discriminated high health problem group from the other groups, and the second the low health problem group from the others (**Fig 1A**).

Pillai's Trace showed significant effect of the % of tree cover on the frequency of stereotype and FCM level (v = 0.429; F (2, 15) = 5.632; p = 0.015). However, a separate univariate ANOVA on the outcome variables revealed significant treatment effect only on frequency of stereotype (F (1, 16) = 6.668; p = 0.020) and not on FCM level (F (1, 16) = 0.012; p = 0.914). MANOVA test followed by discriminate analysis revealed one discriminate function. The correlations between outcomes and discriminant function revealed that the frequency of stereotype loaded fairly evenly with the function (r = 0.745). The FCM values had a correlation of r = -0.032 with the discriminant function. The discriminant function plot showed clear separation of the two tree categories (**Fig 1B**). Similar results were obtained for nature of substrate, presence of stones and keeper attitude (**Table 4 and Fig 1C, 1D & 1E**).

Pillai's Trace showed that enclosure size had significant effect on the frequency of stereotype and FCM level (v = 0.604; F (2, 15) = 11.421; p = 0.001). A univariate ANOVA on the outcome variables revealed a significant treatment effect on the frequency of stereotype (F (1, 16) = 15.491; p = 0.001) and FCM level ((F (1, 16) = 11.877; p = 0.003). The MANOVA test, followed by discriminate analysis, revealed one discriminate function. It explained 100% of the variance, and Canonical R^2^ = 0.777. This discriminant function differentiated the enclosure categories, Ʌ = 0.396, χ^2^ (2) = 13.880, p = 0.001. The correlations between outcomes and discriminant function revealed that the frequency of stereotype loaded fairly evenly with the function (r = 0.797). The FCM values had a correlation of r = 0.698 with the discriminant function. The discriminant function plot showed that it discriminated the two enclosure categories (**Fig 1F**). The MANOVA test showed that variables like health condition, % of tree cover, substrate, presence of stone, keeper attitude and enclosure size have significant effect on both stereotypes and FCM. Further, DFA showed that the health condition and enclosure size of tigers were discriminated well by both stereotypes and FCM levels combined together.

### (5) Relationship between stereotypic behaviour and FCM levels

**Fig 2:**
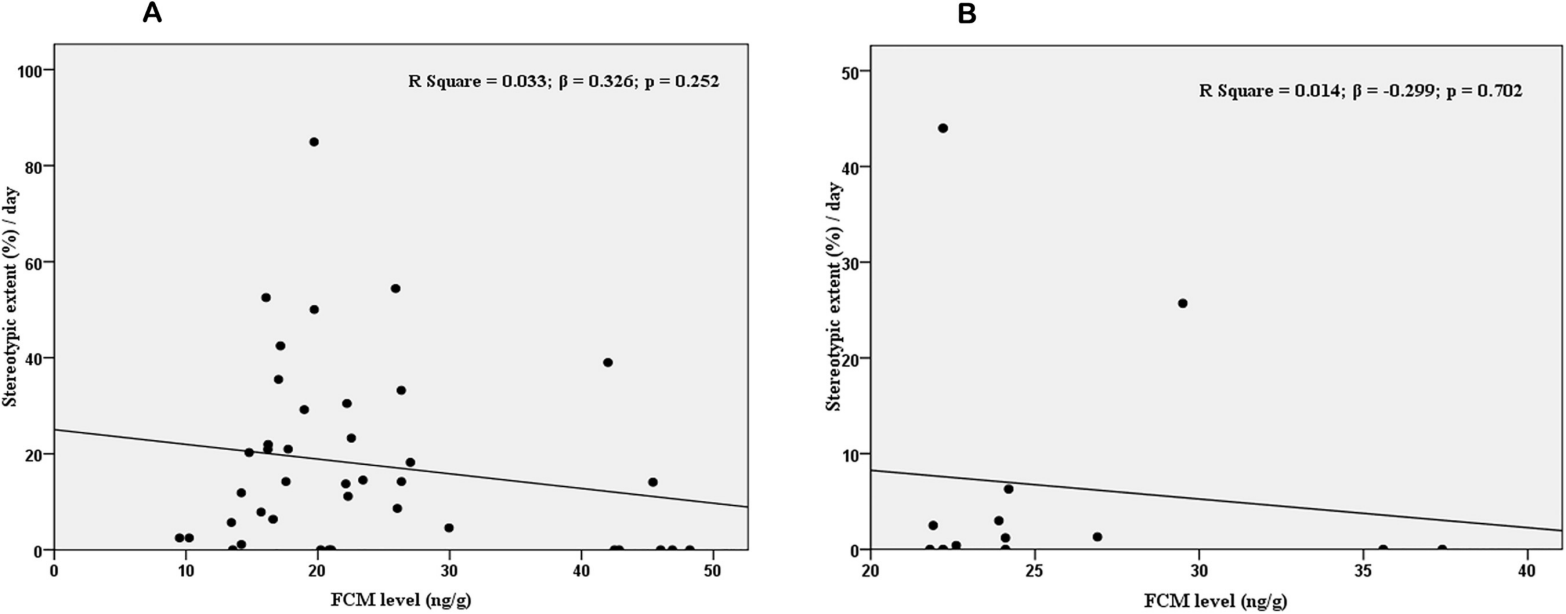
Relationship between FCM and stereotypic behaviour among (2A) tigers and (2B) leopards.

The stereotype behaviour though decreased with FCM level both in tigers and leopards, but not significant both in tigers (R^2^ = 0.033, *B -0*.*3*2*6*, p = 0. 252) (**Fig 2A**) and leopards (R^2^ = 0.014, *B* -0.299, p = 0. 702) (**Fig 2B**). These results suggest that the stereotype levels were not fluctuating exactly in tune with FCM, among both the cats. However, when the effect of health status was controlled using ANCOVA, the stereotype showed significant increase with the FCM level in tigers (Beta for FCM = 0.756, t = 2.426, p = 0.029; F(1,14) = 5.884; p <0.05; ηp^2^ = 0.296). On the other hand in leopards, the stereotype showed significant increase with the FCM level, only when tree cover, den, stone and keeper attitude were controlled (Beta for FCM = 0.348, t = 3.043, p = 0.012; F(1,10) = 9.263; p<0.05; ηp^2^ = 0.481).

### (6) Factors driving the stereotypic extent and FCM levels

#### (a) Stereotype

The binary logistic regression, which predicts the outcome of stereotypic behaviour using biological and environmental factors, showed that psychological enrichments of enclosures, viz., pool and stones, positive keeper attitude, large enclosures and social condition (groups rather than solitary) had significant effect on tigers in the absence of stereotypic behaviour (**Table 5**). These five variables together explain 43% of variations in the presence/absence of stereotypic behaviour among the tigers. In leopards, the origin of the individuals (with wild-born showing more stereotype), age (stereotype more in young individuals), and environmental factors that enrich the enclosures, viz., the presence of pool, den and high frequency of tree cover had significant effect in the absence of stereotypic behaviours. These five variables together explain 81% of variations in the presence/absence of stereotypic behaviour among the leopards sampled.

**Table 5:** Binary logistic regression equation employed to predict the outcome of stereotypic behaviour (0 = absence and 1 presence) and FCM levels (0 = low and 1 high) in tigers and leopards using the biological and environmental variables.

Species	Included	B (SE)	95% CI for odds ratio		
			Lower	Odds ratio	Upper
Tiger Stereotype	Constant	3.777 (1.389)	-	43.67	-
	Pool presence	-2.594* (1.462)	0.004	0.075	1.311
	Stone presence	3.386* (1.769)	0.906	29.028	929.813
	Keeper attitude—positive		-	-	-
	Keeper attitude—neutral	-2.553* (0.891)	0.014	0.078	0.446
	Keeper attitude—negative	17.470* (1.797E4)	0.000	3.865E7	-
	Enclosure—large	-3.412* (1.214)	0.003	0.033	0.356
	Sociality—group	-1.854* (0.672)	0.042	0.157	0.585
	R^2^ = 0.43 (Hosmer and Lemeshow), 0.249 (Cox & Snell), 0.404 (Nagelkerke); Model χ^2^ (6) = 32.630, p<0.001; * significant at p < 0.05				
Leopard Stereotype	Constant	-27.226 (2.556E3)	-	-	-
	Origin—wild	170.979* (1.534E4)	0.000	1.799E74	-
	Pool—absence	55.838* (5.112E3)	0.000	1.778E24	-
	Den—presence	-147.013* (1.739E4)	0.000	0.000	-
	Age	-14.306* (1.278E3)	0.000	0.000	-
	Tree—low %	48.257* (1.329E4)	0.000	9.073E20	-
	R^2^ = 0.81 (Hosmer and Lemeshow), 0.615 (Cox & Snell), 0.824 (Nagelkerke); Model χ^2^ (5) = 47.771, p<0.001; * significant at p < 0.05				
Tiger FCM	Constant	20.180 (1.704E4)	-	-	-
	Health problem—nil	-		5.807E8	-
	Health problem—low	-40.653 * (2.112E4)	0.000	0.000	-
	Health problem—high	-62.755* (2.473E4)	0.000	0.000	-
	Enclosure—small	21.572* (1.248E4)	0.000	2.338E9	-
	R^2^ = 0.64 (Hosmer and Lemeshow), 0.649 (Cox & Snell), 0.909 (Nagelkerke); Model χ^2^ (3) = 23.023, p<0.001; * significant at p < 0.05				
Leopard FCM	Constant	-21.203* (1.340E4)		0.000	-
	Stone—absence	20.797* (1.340E4)	0.000	1.077E9	-
	Keeper attitude—neutral	21.608* (2.842E4)	0.000	2.423E9	-
	R^2^ = 0.94 (Hosmer and Lemeshow), 0.675 (Cox & Snell), 0.95 (Nagelkerke); Model χ^2^ (3) = 17.995, p<0.001; * significant at p < 0.05				

FAMD on variables influencing stereotype of tigers revealed that out of 18 factors tested, 13, viz., enclosure size, tree and grass cover, pool, stone, den, health problem, keeper attitude, age, sociality, temperature regulator, barriers and ambient temperature had high correlation in the first three principal components; these were selected for multiple regression analysis (**Table 6**). The analysis that tests the effect of the 13 independent variables on the dependent (stereotype) variable revealed that small enclosure size, and absence of sociality influenced negatively, while keepers with negative and neutral attitudes influenced positively suggesting the importance of these three factors on the intensity of stereotype among tigers (**Table 7.**). A similar analysis for stereotype data on leopards revealed that age and tree cover availability are the only significant variables influencing the stereotypes. Thus, it is inferred that the absence of pool and stones, negative and neutral keeper attitude, small enclosures and keeping them in solitary condition were the causal factors for the onset of stereotypes in tigers; of these, enclosure size, sociality and keeper attitude caused quantitative (intensity) changes in stereotype among tigers. Absence of pool, den and tree cover are the most important environmental variables influencing the onset of stereotypes significantly in leopards; of these, tree cover had a significant influence on the intensity of stereotype.

**Table 6:** Influence of variables (COS^2^) on different components obtained from Factor Analysis of Mixed Data (FAMD) by FactoMineR to evaluate the effects of biological and environmental variables on stereotype and FCM of tigers and leopards in captivity.

Varaiables	Stereotype						FCM			
	Tiger			Leopard			Tiger		Leopard	
	Components						Components			
	1	2	3	1	2	3	1	2	1	2
**Continuous**										
Age	0.034	**0.470**	0.102	0.000	0.001	**0.509**	0.231	0.223	0.147	**0.577**
Ambient temperature	0.013	0.049	**0.465**	0.025	0.055	0.109	0.043	0.288	0.127	0.127
Visitor number	0.047	0.147	0.304	0.011	0.081	0.009	0.091	0.138	0.041	0.082
Enclosure size	**0.467**	0.048	0.008	0.125	**0.612**	0.004	**0.476**	0.095	**0.893**	0.088
Sociality	0.202	**0.636**	0.008	**0.791**	0.104	0.020	**0.485**	0.114	**0.905**	0.076
Grass cover	**0.861**	0.008	0.024	**0.876**	0.010	0.022	**0.783**	0.104	**0.905**	0.076
Tree cover	**0.919**	0.020	0.002	**0.901**	0.029	0.015	**0.833**	0.019	**0.905**	0.076
**Categorical**										
Sex female	0.259	0.122	0.142	0.365	0.003	0.027	0.124	0.243	**0.79**	0.157
Sex male	0.259	0.122	0.142	0.365	0.003	0.027	0.124	0.243	**0.79**	0.157
Origin wild	0.120	0.111	0.330	**0.695**	0.163	0.061	0.003	**0.908**	**0.79**	0.157
Origin zoo	0.120	0.111	0.330	**0.695**	0.163	0.061	0.003	**0.908**	**0.79**	0.157
Substrate artificial	0.292	0.026	0.300	**0.574**	0.172	0.032	**0.803**	0.102	**0.589**	0.318
Substrate natural	0.292	0.026	0.300	**0.574**	0.172	0.032	**0.803**	0.102	**0.589**	0.318
Pool absence	**0.626**	0.000	0.009	0.051	**0.707**	0.035	**0.36**	0.011	-	-
Pool presence	**0.626**	0.000	0.009	0.051	**0.707**	0.035	**0.36**	0.011	-	-
Stone absence	**0.948**	0.000	0.008	**0.687**	0.202	0.009	**0.936**	0.041	**0.947**	0.030
Stone presence	**0.948**	0.000	0.008	**0.687**	0.202	0.009	**0.936**	0.041	**0.947**	0.030
Den absence	**0.912**	0.015	0.011	**0.700**	0.002	0.128	**0.862**	0.049	**0.884**	0.039
Den presence	**0.912**	0.015	0.011	**0.700**	0.002	0.128	**0.862**	0.049	**0.884**	0.039
Temperature regulator absence	0.345	**0.517**	0.028	-	-	-	-	-	-	-
Temperature regulator presence	0.345	**0.517**	0.028	-	-	-	-	-	-	-
Barrier absence	0.391	**0.482**	0.005	0.100	**0.611**	0.001	-	-	-	-
Barrier presence	0.391	**0.482**	0.005	0.100	**0.611**	0.001	-	-	-	-
Breed absence	0.034	0.087	0.077	0.107	0.153	**0.704**	0.0207	**0.511**	0.0545	**0.837**
Breed presence	0.034	0.087	0.077	0.107	0.153	**0.704**	0.0207	**0.511**	0.0545	**0.837**
Health problem high	**0.926**	0.010	0.007	**0.629**	0.253	0.065	**0.897**	0.000	**0.718**	0.237
Health problem low	0.122	0.013	0.394	**0.787**	0.038	0.106	**0.643**	0.124	**0.979**	0.016
Health problem no	**0.739**	0.029	0.065	0.085	0.131	**0.670**	**0.382**	0.178	0.1697	**0.750**
Keeper attitude negative	0.158	0.212	**0.415**	-	-	-	-	-	**0.710**	0.141
Keeper attitude neutral	**0.521**	0.086	0.008	0.256	0.346	0.039	**0.933**	0.017	-	-
Keeper attitude positive	0.381	0.019	0.113	0.256	0.346	0.039	**0.933**	0.017	**0.710**	0.141
Eigenvalue	5.7	2.8	2.3	5.18	3.11	2.32	6.3	2.7	7.8	3.6
% of variance	27.4	13.5	11.0	28.8	17.3	12.9	36.8	16.0	48.9	22.4
Cum. % of variance	27.4	40.9	51.8	28.8	46.1	59.0	36.8	52.9	48.9	71.3

(Variables with COS^2^ values ≥ 0.4 (bolded) were selected for further multiple regression analysis).

**Table 7:** Regression equation model to explore the effect of biological and environmental factors on the intensity of stereotype and FCM level among tigers and leopards at six Indian zoos.

Species	Factors	Un-standardized Coefficients		Std. Coefficients	t	Sig.	F	p	R^2^
		B	Std. Error	Beta					
Tiger Stereotype	(Constant)	-9.24	4.533		-2.038	0.04	22.51	0.000	0.45
	Enclosure size	-13.411	2.733	-0.385	-4.907	0.00			
	Sociality	-5.787	2.783	-0.159	-2.08	0.04			
	Keeper neutral	13.852	2.597	0.397	5.334	0.00			
	Keeper negative	15.397	6.322	0.183	2.436	0.02			
Leopard Stereotype	(Constant)	19.243	2.978		6.461	0.000	13.31	0.000	0.61
	Age	-0.651	0.214	-0.354	-3.045	0.004			
	Tree cover	-10.107	2.844	-0.413	-3.554	0.001			
Tiger FCM	(Constant)	36.214	2.567		14.108	0.000	16.11	0.000	0.46
	Health problem low	-15.007	3.476	-0.64	-4.318	0.000			
	Health problem high	-17.312	3.119	-0.823	-5.551	0.000			
Leopard FCM	(Constant)	37.4	2.519		14.848	0.000	19.09	0.000	0.52
	Keeper positive	-11.938	2.732	-0.717	-4.37	0.000			

#### (b) FCM

The binary logistic regression to predict the outcome of FCM levels (0 = low; 1 = high) using biological and environmental variables showed that health status in biological variables and enclosure size among environmental factors had significant effect among tigers (**Table 5**). The FCM level decreased with health problems of individuals (from individuals with no major health problem → high → low) and among individuals in larger enclosures compared to those in smaller enclosures. These two variables together explain 64% of variations in FCM level among the tigers sampled. On the other hand, in leopards, the presence of stones and keepers’ attitude had significant effect on FCM levels. The FCM level was significantly lower among leopards in enclosures with the presence of stones than those with their absence and among leopards under positive keepers' attitude than those with neutral attitude. These two variables together explain 94% of variations in FCM level among the leopards sampled.

FAMD on variables influencing FCM level of tigers showed that of the 17 variables tested, 12, viz., enclosure size, sociality, grass and tree cover, origin, substrate, stone, den, breeding health problem and keeper attitude had higher correlation with the first two principal components (**Table 6**); these components were selected for further multiple regression analysis. The resulting equations showed that among the variables, health status is the significant variable among tigers and keepers’ attitude the significant variable among leopards, influencing the FCM levels in the two cats (**Table 7.**).

## Discussion

### i) Stereotypic behaviour in tigers

Binary logistic regression results showed that among the various biological and environmental factors assessed, large enclosures, presence of pool, and stones, positive keepers’ attitude and keeping of tigers in social environment, i.e., with conspecifics, were the best predictors for the prevention of stereotype among tigers in captivity. Results of multiple regression analysis also showed that enclosure size, sociality and keepers' attitude are the best predictors of quantitative changes in stereotype of tigers

#### a) Influence of enclosure size and its enrichments on stereotype

The size of the enclosure is one of the most influencing factors of stereotypic behaviour, as tigers housed in enclosures smaller than the size prescribed by CZA stereotyped higher than those in larger enclosures. In line with the present study, other captive studies [54–55, 11, 56] also showed that tigers housed in larger enclosures are more active than those placed in smaller ones. Restricted movement due to space limitations was considered as one of the primary contributors to captivity-induced stress [57–58], and earlier studies on abnormal behaviour of captive animals also supported this observation [59]. [56], who quantified the influence of enclosure size on distance covered and paced (stereotyped) by tigers in 14 enclosures of size ranging from 21 to 35865 m^2^, found that among eight of them with less than 1000 m^2^ area, the enclosure size was negatively linked to pacing. This finding is in agreement with [60] as well. Given their large body mass and higher energetic requirements, tigers range widely under natural conditions and thus are regarded as wide-ranging species [61–62]. Tigers, with their nature of following their dispersing prey, are great wanderers, roaming from place to place in a radius of 24–32 km [63], and travel 5–30 km per day for food and even 50–60 km under unusual circumstances [64]. Home-ranges of tigers are vary from 16 km^2^ in the alluvial flood plains of the Indian subcontinent to several hundreds of square kilometres in the cold climes of the Russian Far East [65–67, 61]. [68] reported that tiger range might be as high as 1500 square miles. Thus, home range patterns of tigers can be informative of their space requirements [69] and so, to elicit their natural behaviour, [56] recommend building or modifying the existing enclosures to provide over 1000 m^2^ area. [70] found an association between enclosure size and successful reproduction in felids. However, [54] and [71] did not find total size of enclosure a major factor in pacing. Indeed, [71] consider that enclosure size is not a significant factor if it is not barren, and the quality of space in terms of complexity is more important than its quantity. [54] and [72] also arrived at such a similar conclusion earlier. [73] states that naturalistic enclosures result in a more naturalistic behaviour. [74] also recommend the availability of ample retreat and hiding space for felids. Hence, it is assumed that both large and enriched naturalistic enclosures are essential to prevent the onset of stereotypes among tigers in captivity.

The present study also supports this view, as in addition to large enclosure size, quality of enrichments, viz., the presence of pool and stone could significantly influence stereotypes among tigers in captivity. The presence of a pool with clear water will increase exploratory behaviour and reduce stereotypic pacing in tigers [75] and is indicative of their enhanced welfare [71]. "Tiger being an emigrant from cool northern climes, centuries of acclimatization have left the tiger still intolerant to tropical heat. To escape the heat, it takes shelter in caves or covers and many tigers, particularly during the hot weather, walk to water and lie in it during the sweltering hours of the day and presence of water is an important component of its natural habitat for quenching the thirst" [63]. Since tigers in the wild like water and can swim for kilometres [76–77], using a pool in a captive environment can elicit their natural behaviour. Indeed, many international guidelines recommend water pools as a structural feature in tiger enclosures [78–80].

#### b) Influence of sociality on stereotype

Our study shows that tigers managed in social conditions stereotyped lower than those managed in solitary condition. [81] and [54] also reported that social interaction results in the reduction or absence of pacing. However, this is intriguing, as several species of felids are solitary living with vast territories in the wild [82], and considering the territorial behaviour of tigers, especially of males, in their natural habitats. [68] explained this contradiction citing the composition of a large group of seven individuals by [83], who stated that "the presumed territoriality system of male tigers appears to be less rigid than that, for example, of many antelopes and birds' and "it is possible that territorial behaviour may be modified under certain environmental conditions such as shortage of water or cover". Further, [68] stated that female in oestrus may travel widely and is sometime followed by several males. Studies across a number of other species have also demonstrated social isolation to be associated with high levels of stereotypic behaviour and chronic stress [84–87]. On the other hand, [88] state that felid species, which are generally solitary in the wild, are in pairs or trios in zoos due to constraints of space and as a result, although arguably a source of social enrichment, can also be a source of chronic stress [3] and can affect reproduction too [89–90]. However, the present study shows that keeping the tigers in sociality might positively influence their welfare in captivity.

#### c) Influence of keeper’s attitude on stereotype

Our study shows that positive keeper attitude is a predictor for the absence of stereotype among the tigers in captivity and is a significant part of the individual animal's environment in captivity. Similar to other environmental factors like large enclosures and enrichments that assist in lowering stereotypes significantly, tigers under keepers with positive attitude show significantly lesser stereotype than those under keepers with negative and neutral attitude. According to [91], the principal goal of environmental enrichment is the identification and provision of appropriate stimuli necessary for physiological and psychological well-being of a wide range of captive species [37]. This broad definition of environmental enrichment can be extended to human–animal relationships (HAR), such as keeper attitude in captive environment as well. According to [92], the HAR in captivity is characterised in three ways, viz., negative, neutral and positive relationships, with positive HAR potentially encouraging species-specific behaviours meeting the same goals as ‘traditional’ forms of enrichment. As stereotypical behaviour associated with stress has implications for both captive wellbeing and breeding, reducing this through environmental enrichment, such as the HAR (positive keeper attitude) is important. [91] suggests that HAR might have significant effect on animal welfare, possibly as enrichment.

### ii) Stereotypic behaviour in leopards

Interestingly, for leopards, the enclosure size did not enter into the binary logistic or multiple regressions to predict stereotypes. This could be due to, that unlike tigers, which are sensitive to human disturbances and largely restricted to wildlife reserves [93], leopards, being the most adaptable and versatile large carnivores [94], can manage in smaller areas with dense undergrowth in human-dominated landscapes [95–96]. Our study also shows that stereotypes in leopards decreased significantly with the presence of pool, and den and high density of tree cover in the enclosure; it however increased with younger and wild-born leopards, these factors accounting for 81% of variation in stereotypic prevalence, as inferred from binary logistic regression. Multiple regression also showed that tree cover reduced the stereotypic behaviour in leopards significantly. Factors like the presence of pool and den, tree cover are the characteristic features of leopards' natural habitat, with which the species in the wild often associate. For example, leopards use trees and rocky areas for resting and hide their hunted prey on tree branches to avoid disturbance from carrion feeders [63]. Thus, the results show the significance of greater enrichments simulating natural environments for leopards' psychological welfare over increasing the size of enclosures [97]. Another factor that entered into the binary logistic regression to predict stereotype in leopards is their origin. Among the leopards observed in the present study, 81% were wild-born, with majority (69%) arriving at the zoo when young (<1 year) and most of which (67%) were managed in solitary condition. Thus, the significance and higher levels of stereotypes observed in the present study among wild-origin and younger age-class leopards could be due to loss of maternal association and or isolation from conspecifics at the zoos. The deprivation of socialisation process especially in early life could reduce adaptability [98] and promote stereotypes [99]. [100] also suggested that enrichment would be highly positive for younger individuals.

### iii) FCM level in tigers and leopards

In the binary logistic regressions, enclosure size and health status are considered significant variables influencing FCM level in tigers, while the presence of stone and keeper attitude significantly influences the FCM levels in leopards. Multiple regression equations of FCM also corroborated the same, that is, the health status and keeper attitude influence the FCM levels of the felids most significantly and quantitatively. We have already discussed the role of size of enclosure in relation to stereotypic response of tiger. Being a wide-ranging species, the spatial constraints in captivity have increased its pacing significantly [56], and, as a consequence, stress level was elevated in captivity [57, 101]. [102] also observed elevated faecal corticosterone when Tigrina (*Leopardus tigrinus*) and Margay (*Leopardus wiedii*) were in smaller enclosures. As such, the significant increase in FCM levels among tigers managed in smaller enclosures in the present study might be a consequence of stress due to spatial constraints. Tigers without health problems showed higher levels of FCM indicating that healthy tigers experience higher stress. Tigers without health problems in the present study consisted mostly of middle age-class. Individuals at this stage in wild condition tend to disperse and establish territory and as a consequence are more likely to exhibit extensive movements than the other age-classes (young and older). As space is a major limiting factor in captive conditions, middle-aged tigers, which are free of health problems, are likely to have higher levels of stress and higher FCM as shown in the present study.

This study showed that the keeper’s attitude is an important predictor of FCM levels in leopards (**Tables 5 & 7**), as positive keeper attitude significantly reduced the level among leopards than those under neutral attitude **Table 3**. Earlier, positive correlation was reported between the quality of keeper interactions, and increased reproductive success, in various species of small exotic felids [90]. [47] in their study on clouded leopards (*Neofelis nebulosa*) housed in different zoos also found a negative correlation between faecal corticoid levels and the amount of time primary caretakers spent with the animals (positive keeper attitude), while a positive correlation was observed between cortisol levels and the number of keepers (negative effects of keepers). An interesting result of the present study is that the provision of stone as a psychological enrichment is a significant predictor of high FCM levels in leopards (**Table 5**). A review and meta-analyses of literature [20] also indicated that enrichment is a successful technique in reducing stress in zoo animals. [103] also reported a causal relationship between the provision of additional hiding spaces and a decline in faecal corticoid concentration in clouded leopards.

### iv) Relationship between stereotypic behaviour and FCM levels

Although not all stereotypic behaviours are a response to stress [84], their occurrence among captive animals is usually associated with elevated cortisol levels [9, 26], and a high corticoid variability is particularly thought to be an indicator of chronic or ‘‘bad” stress [27]. However, our study reveals an insignificant relationship between levels of stereotypic behaviour and FCM. [15] also found that the serum cortisol concentrations varied among individuals and did not clearly correspond with the expression of stereotypic swaying in captive African elephants (*Loxodonta africana*). Other studies also reported inconsistent association between these two measures, as some animals that exhibited high rates of stereotypic behaviour also had high glucocorticoid concentrations [104–105, 47, 106–107], and yet others that exhibited high rates of stereotypic behaviour had lower cortisol concentrations [9]. [108] found on repeated immobilization experiments that minks in non-stress situation to have lower baseline cortisol levels, and higher cortisol responses when immobilised (stressed), and concluded that under non-stress conditions, stereotypes and physiological stress parameters may be negatively correlated, while exposure to stress brings about a positive correlation.

[28] state that a host of biological variables may have confounding effect on Faecal Glucocorticoid Metabolite (FGM) measures, viz., length of time in captivity [109], normal daily changes [110–111] and normal seasonal changes in glucocorticoid excretion [112–114], reproductive status [114], sex [115], body condition [116] and animal diet [117] to cite a few, and might influence the interpretation of an animal's response to various stressors. Indeed, our results of ANCOVA also revealed that variables viz., health status in tigers and tree cover, den, stone and keeper attitude in leopards had confounding effects on the relationship between FCM and stereotype. Further, [28] also state that lack of available seasonal FCM values and knowledge of 'normal' values makes it difficult to assess whether FCM levels are elevated in relation to biological distress. Moreover, different steroid hormones are metabolized and excreted in various proportions via urine and/or faecal matter [118–119] and hence relative proportions of glucocorticoid metabolites should be measured to make meaningful comparisons. Thus, in the existing literature, information on the range of FGM concentrations that are deleterious to the animals is a largely unresolved issue, impeding the utility of FGM assays in conservation biology and other fields. However, one must also remember that elevated FGM concentrations do not automatically indicate a state of distress [120–121]. Glucocorticoids are adaptive mediator of the stress response and help the animals to redirect activities [122], playing a crucial role in glucose homeostasis and suppression of stimulation of other body responses (e.g., immunological) that prevent damage to the body [123, 24] and serve as a forewarning of a possible harm [124]. Further, [125] found that the temporal aspect (short- or long-term) of experience might influence the direction of stress effect. Thus, if the same stressor continues, an individual with higher stereotype and lower FCM might change into the other category, i.e., a low stereotype with high FCM. Hence, one needs caution in interpreting variations in FCM levels, as they need not always reflect the current status of stress in an animal. So, we suggest that rather than FCM level, behaviours such as stereotype should be considered as a better stress/welfare indicator, as it reflects an animal’s first attempt to cope with a stressor and indicate at an early stage when welfare is at risk [126].

## Conclusions and management recommendations

We conclude that: (i) Large enclosures with psychological enrichments, viz., pool (with clean water) and stones, positive keeper attitude and proper health care are essential for tigers, and (ii) Enclosure enrichments with abundant tree cover, presence of pool, stones, and den, and positive keeper attitude are important for leopards, to ensure their welfare and visitors' satisfaction of seeing them behave naturally in captivity. The results of the present study could help to refine captive protocols for big cats among zoos, where similar conditions prevail. We suggest the following remedial measures:

- Tigers are wide-ranging in nature, and stereotype and stress decrease with enclosure size. Hence, to reduce their stereotype, especially in middle-aged class, where it is the highest (i) the existing zoos with enclosures smaller than suggested by CZA need to enlarge their size, wherever possible, (ii) middle-aged tigers be provided with the largest enclosures available at the zoo, and (iii) since felids are crepuscular and nocturnal, the existing zoos need to enlarge their night cells as well, if possible, and new zoos have to design larger ones.
- The outdoor enclosures of both tigers and leopards should be enriched with higher tree cover, presence of pool (with clear water), stones, and den (mimicking their natural habitats) and should be provided with conspecific socialisation to reduce stress and promote naturalistic behaviour among them in captivity.
- To overcome the problem of development of stereotypic behaviour, the younger age-class individuals, especially those brought from wild as orphan, should be kept with similar age-mates and also with free access to conspecifics.
- Keeper attitude is an important factor to reduce stereotypes and stress both in tigers and leopards, and thus keepers should be scientifically trained and counselled for better upkeep of big cats in captivity.

## Supporting information

S1 Table Details of individual tigers and leopards sampled for stereotype, and FCM in the present study, their characteristics and enclosure size. (DOCX)
