# Supplementary material for: Prevalence and determinants of stereotypic behaviours and physiological stress among tigers and leopards in Indian zoos
Source: PLoS One. 2017 Apr 17;12(4):e0174711. doi: 10.1371/journal.pone.0174711 (PMC5393558; doi:10.1371/journal.pone.0174711)
Supplement: S1 Table — (DOCX) [file pone.0174711.s001.docx]

S1 Table: Details of individuals tigers and leopards sampled for stereotype, and FCM in the present study, their characteristics and enclosure size.

| **Place^a^** | **Tiger ID** | **Age - Sex** | **Origin** | **Enclosure size m^2^** | **Sampled for (Stereo : FCM)** | **Leopard ID** | **Age - Sex** | **Origin** | **Enclosure size m^2^** | **Sampled for (Stereo : FCM)** |
| --- | --- | --- | --- | --- | --- | --- | --- | --- | --- | --- |
| Zoo 1 | T1 | 12 - M | Zoo | 2373.67 | Yes : Yes | L1 | 08 - F | Zoo | 195.93 | Yes : Yes |
| Zoo 1 | T2 | 10 - F | Zoo | 320.08 | Yes : Yes | L2 | 02 - F | Zoo | 195.93 | Yes : Yes |
| Zoo 1 | T3 | 10 - M | Zoo | 83.61 | Yes : Yes | L3 | 20 - F | Zoo | 195.93 | Yes : Yes |
| Zoo 1 | T4 | 07 - M | Zoo | 40.59 | Yes : No | L4 | 14 - M | Wild | 195.93 | Yes : Yes |
| Zoo 1 | T5 | 07 - F | Zoo | 29.58 | Yes : Yes | L5 | 07 - M | Wild | 21.18 | Yes : Yes |
| Zoo 1 | T6 | 07 - M | Zoo | 83.61 | Yes : Yes | - | - | - | - | - |
| Zoo 1 | T7 | 08 - F | Zoo | 220.08 | Yes : Yes | - | - | - | - | - |
| Zoo 2 | T8 | 08 - F | Wild | 118.91 | Yes : Yes | L6 | 20 - M | Wild | 26.57 | Yes : Yes |
| Zoo 2 | T9 | 16 - M | Wild | 118.91 | Yes : Yes | L7 | 05 - M | Wild | 23.41 | Yes : Yes |
| Zoo 2 | T10 | 12 - M | Zoo | 225.75 | Yes : Yes | L8 | 01 - F | Zoo | 26.57 | Yes : Yes |
| Zoo 2 | - | - | - | - | - | L9 | 06 - M | Wild | 23.41 | Yes : No |
| Zoo 3 | T11 | 10 - M | Zoo | 1481.20 | Yes : Yes | L10 | 21 - M | Wild | 35.11 | Yes : Yes |
| Zoo 3 | T12 | 10 - F | Zoo | 1481.20 | Yes : Yes | L11 | 10 - F | Wild | 32.6 | Yes : No |
| Zoo 3 | T13 | 06 - F | Zoo | 263.56 | Yes : No | L12 | 10 - M | Wild | 1234.06 | Yes : No |
| Zoo 3 | T14 | 04 - M | Zoo | 346.10 | Yes : Yes | - | - | - | - | - |
| Zoo 3 | T15 | 04 - F | Zoo | 1481.20 | Yes : Yes | - | - | - | - | - |
| Zoo 3 | T16 | 03 - M | Zoo | 1481.20 | Yes : Yes | - | - | - | - | - |
| Zoo 3 | T17 | 03 - M | Zoo | 1481.20 | Yes : Yes | - | - | - | - | - |
| Zoo 3 | T18 | 03 - F | Zoo | 1481.20 | Yes : Yes | - | - | - | - | - |
| Zoo 3 | T19 | 01 - F | Zoo | 1481.20 | Yes : Yes | - | - | - | - | - |
| Zoo 3 | T20 | 01 - F | Zoo | 1481.20 | Yes : No | - | - | - | - | - |
| Zoo 3 | T21 | 01 - M | Zoo | 1481.20 | Yes : No | - | - | - | - | - |
| Zoo 3 | T22 | 01 - M | Zoo | 263.56 | Yes : No | - | - | - | - | - |
| Zoo 3 | T23 | 01 - M | Zoo | 263.56 | Yes : No | - | - | - | - | - |
| Zoo 3 | T24 | 03 - F | Zoo | 5187.00 | Yes : No | - | - | - | - | - |
| Zoo 3 | T25 | 03 - F | Zoo | 5187.00 | Yes : No | - | - | - | - | - |
| Zoo 3 | T26 | 03 - F | Zoo | 5187.00 | Yes : No | - | - | - | - | - |
| Zoo 3 | T27 | 02 - F | Zoo | 5187.00 | Yes : No | - | - | - | - | - |
| Zoo 3 | T42 | 12 - M | Zoo | 263.56 | No : Yes | - | - | - | - | - |
| Zoo 4 | T28 | 15 - F | Wild | 1587.72 | Yes : No | L13 | 21 - M | Wild | 119.5662 | Yes : No |
| Zoo 4 | T29 | 21 - F | Zoo | 1587.72 | Yes : No | L14 | 03 - M | Wild | 193.1454 | Yes : No |
| Zoo 4 | T30 | 04 - M | Zoo | 1587.72 | Yes : No | - | - | - | - | - |
| Zoo 4 | T31 | 07 - M | Wild | 24.38 | Yes : No | - | - | - | - | - |
| Zoo 4 | T32 | 02 - M | Zoo | 1568.49 | Yes : No | - | - | - | - | - |
| Zoo 4 | T33 | 08 - M | Zoo | 1568.49 | Yes : No | - | - | - | - | - |
| Zoo 4 | T34 | 01 - M | Zoo | 1568.49 | Yes : No | - | - | - | - | - |
| Zoo 4 | T35 | 08 - F | Zoo | 1884.78 | Yes : No | - | - | - | - | - |
| Zoo 4 | T36 | 07 - M | Zoo | 1884.78 | Yes : No | - | - | - | - | - |
| Zoo 5 | T37 | 14 - M | Wild | 2620.00 | Yes : No | L15 | 08 - M | Wild | 851 | Yes : No |
| Zoo 5 | T38 | 14 - F | Wild | 2620.00 | Yes : No | L16 | 15 - F | Wild | 851 | Yes : No |
| Zoo 5 | T39 | 12 - M | Zoo | 3629.00 | Yes : No | L17 | 13 - F | Wild | 851 | Yes : No |
| Zoo 5 | T40 | 12 - F | Zoo | 3629.00 | Yes : No | - | - | - | - | - |
| Zoo 5 | T41 | 12 - F | Zoo | 3629.00 | Yes : No | - | - | - | - | - |
| Zoo 6 | - | - | - | - | - | L18 | 10 - F | Wild | 300 | Yes : No |
| Zoo 6 | - | - | - | - | - | L19 | 05 - F | Wild | 300 | Yes : No |
| Zoo 6 | - | - | - | - | - | L20 | 16 - F | Wild | 74.32 | Yes : No |
| Zoo 6 | - | - | - | - | - | L21 | 14 - M | Wild | 74.32 | Yes : No |

^a^ For confidential reasons, the six zoos are labelled by a number, from 1 to 6.
